# The genome of the deep-sea anemone *Actinernus* sp. contains a mega-array of ANTP-class homeobox genes

**DOI:** 10.1098/rspb.2023.1563

**Published:** 2023-10-25

**Authors:** Sean Tsz Sum Law, Yifei Yu, Wenyan Nong, Wai Lok So, Yiqian Li, Thomas Swale, David E. K. Ferrier, Jianwen Qiu, Peiyuan Qian, Jerome Ho Lam Hui

**Affiliations:** ^1^ School of Life Sciences, Simon F.S. Li Marine Science Laboratory, State Key Laboratory of Agrobiotechnology, Institute of Environment, Energy and Sustainability, The Chinese University of Hong Kong, Hong Kong, People's Republic of China; ^2^ Dovetail Genomics, LLC, Scotts Valley, CA 95066, USA; ^3^ The Scottish Oceans Institute, Gatty Marine Laboratory, School of Biology, University of St. Andrews, St. Andrews, UK; ^4^ Southern Marine Science and Engineering Guangdong Laboratory (Guangzhou), Guangzhou, People's Republic of China; ^5^ Department of Biology, Hong Kong Baptist University, Hong Kong, People's Republic of China; ^6^ Department of Ocean Science, The Hong Kong University of Science and Technology, Hong Kong, People's Republic of China

**Keywords:** adaptations, deep-sea, cnidarian, genome, homeobox, circadian

## Abstract

Members of the phylum Cnidaria include sea anemones, corals and jellyfish, and have successfully colonized both marine and freshwater habitats throughout the world. The understanding of how cnidarians adapt to extreme environments such as the dark, high-pressure deep-sea habitat has been hindered by the lack of genomic information. Here, we report the first chromosome-level deep-sea cnidarian genome, of the anemone *Actinernus* sp., which was 1.39 Gbp in length and contained 44 970 gene models including 14 806 tRNA genes and 30 164 protein-coding genes. Analyses of homeobox genes revealed the longest chromosome hosts a mega-array of Hox cluster, HoxL, NK cluster and NKL homeobox genes; until now, such an array has only been hypothesized to have existed in ancient ancestral genomes. In addition to this striking arrangement of homeobox genes, analyses of microRNAs revealed cnidarian-specific complements that are distinctive for nested clades of these animals, presumably reflecting the progressive evolution of the gene regulatory networks in which they are embedded. Also, compared with other sea anemones, circadian rhythm genes were lost in *Actinernus* sp., which likely reflects adaptation to living in the dark. This high-quality genome of a deep-sea cnidarian thus reveals some of the likely molecular adaptations of this ecologically important group of metazoans to the extreme deep-sea environment. It also deepens our understanding of the evolution of genome content and organization of animals in general and cnidarians in particular, specifically from the viewpoint of key developmental control genes like the homeobox-encoding genes, where we find an array of genes that until now has only been hypothesized to have existed in the ancient ancestor that pre-dated both the cnidarians and bilaterians.

## 1. Introduction

Deep-sea hydrothermal vents and seeps are characterized by darkness, high hydrostatic pressure and the presence of reducing chemicals such as hydrogen sulfide and methane that serve as energy sources to fuel chemosynthesis. These habitats provide a dramatically different ecological niche from those that rely on photosynthesis for primary production, and as such provide us with very distinctive ecological and evolutionary systems to those commonly studied. Our knowledge of metazoan genomic adaptations to these extreme environments has mainly derived from genome analyses of annelids [1], crustaceans [2], molluscs [3–7], echinoderms [8,9] and fishes [10–12]. The phylum Cnidaria contains over 10 000 species of animals including sea anemones, corals and jellyfishes, and its members play important ecological roles in both shallow-water and deep-sea habitats throughout the world. Nevertheless, there are currently only a limited number of deep-sea cnidarian genomes sequenced, including one from a deep-sea coral [13] and one from a deep-sea anemone [14], both at draft genome qualities, hindering the understanding of how cnidarians adapt to this distinctive, extreme environment.

Higher quality genome assemblies allow analyses of genome architecture and much better inferences of ancestral states and the evolutionary dynamics governing genome organization. The homeobox-encoding genes are excellent markers for analyses of genome architecture due to their abundance in animal genomes and a classification scheme that enables detection of classes, families and orthologues across wide spans of the animal kingdom [15,16]. Given the dramatic expansion in homeobox families early in animal evolution, particularly in the ANTP-class, it is assumed that the predominant mode of their origin was via tandem duplication. This led to an influential hypothesis for the origin of the ANTP-class homeobox genes via a Mega-cluster [17]. Evidence supporting this hypothetical Mega-cluster comes from comparisons of various extant animal genomes in which subsets of ANTP-class genes are still found to be linked, such as the Hox cluster and various families of Hox-linked (HoxL) genes, the NK cluster and NK-linked (NKL) genes and other families like the ParaHox and NK2 genes. However, in bilaterians there is a tendency to find the splits amongst subsets of these genes onto distinct chromosomes are in similar places [18,19]. This implies that the last common ancestor of the bilaterians already had these ANTP-class families distributed across at least four chromosomes, which raises the possibility that the ANTP-class Mega-cluster never actually existed after all. Outgroups of the bilaterians, such as cnidarians, are thus a key group of animals to further test the plausibility of the Mega-cluster hypothesis as high-quality genome assemblies become available.

*Actinernus* is a genus of deep-sea anemones currently represented by six species distributed in the Southern Ocean, Indian Ocean, Atlantic Ocean and Pacific Ocean [20–23]. However, molecular data in this genus is scarce. To date, little is known about the phylogeny of *Actinernus* as only several mitochondrial and ribosomal gene fragments are available from two species, *A. robustus* and *A. elongatus* [23,24], let alone further molecular biological information in these deep-sea species. Here, we assembled and analysed the high-quality genome of a deep-sea anemone *Actinernus* sp. sampled from the South China Sea to deepen our understanding on its genomic architecture. Our analyses revealed a striking finding of a mega-array of ANTP-class homeobox genes that may well reflect an ancient ancestral state for these key developmental control genes, alongside likely molecular adaptations to the deep-sea environment.

## 2. Results and discussion

**Figure 1.**
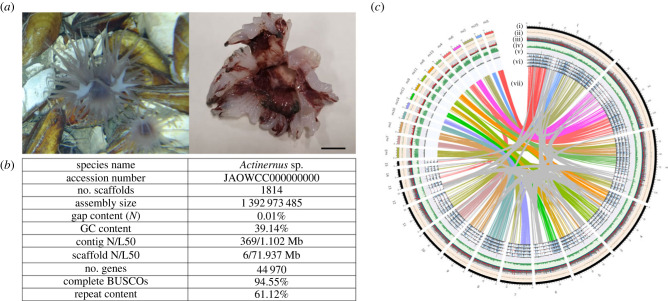
(*a*) Photographs of the *Actinernus* sp. on site (left) and the sampled specimen (right, scale bar = 1 cm); (*b*) genome statistics of *Actinernus* sp.; (*c*) circos plot of the genome assembly of *Actinernus* sp. in comparison with *Nematostella vectensis*, with tracks (i–vii) specified as follows: (i) chromosomes; (ii) GC content percentage (greater than 41.9% in green; less than 37.7% in red); (iii) distribution of repeat elements where DNA transposons, LTRs, LINEs, SINEs, other repeats and unclassified repeats are coloured in red, green, blue, purple, pink and grey, respectively; (iv) gene density; (v) exon density; (vi) transcriptome reads coverage of trunk and tentacle samples; (vii) syntenic blocks showing synteny conservation with *N. vectensis* in colours and self-syntenic regions in grey. All tracks were plotted with 100k window size except tracks (iv) and (v) where 200k window size was used.

The deep-sea anemone *Actinernus* sp. individuals were collected from the Haima cold seep in the South China Sea at a depth of 1386 m (figure 1*a*), where *Actinernus* sp. was commonly observed on the shells of the deep-sea clam *Archivesica marissinica* around cold-seep mussel beds [20,25,26]. The genome of *Actinernus* sp. was sequenced and assembled with PacBio HiFi reads followed by further scaffolding with Omni-C reads (electronic supplementary material, S1 and S2). The genome assembly was 1.39 Gbp with a contig N50 of 1.1 Mbp and scaffold N50 of 71.9 Mbp (figure 1*b*). This high physical contiguity was matched by high completeness, with 94.55% complete BUSCO score (metazoa_genes version odb10) (figure 1*b*; electronic supplementary material, S3). The genome contained 44 970 gene models including 14 806 tRNA genes and 30 164 protein-coding genes, and the majority of the sequences assembled (approx. 80%) were anchored to 15 linkage groups (electronic supplementary material, figure S1), which we labelled according to descending size (figure 1*c*).

Macrosynteny analyses revealed that the genome of *Actinernus* sp. comprised a unique chromosome organization. For instance, the longest chromosome, chr 1, corresponded to at least three chromosomes in other animals while chr 2, 3, 4, 5 and 7 shared syntenic regions with at least two chromosomes (figure 1*c*; electronic supplementary material, figures S2 and S3). Furthermore, 129 self-syntenic blocks (SSBs) were identified, comprising 974 gene pairs across approximately 208 Mbp (approx. 19%) of the 15 chromosomes (electronic supplementary material, figure S4 and S4). Moreover, transposable elements (TEs) accounted for 61.12% of the genome, which was dominated by unclassified TEs (39.05%) with recent expansion activity (electronic supplementary material, figure S5). The burst of TEs was significantly correlated to the large genome size in *Actinernus* sp., as compared with genomes of other cnidarians, deep-sea animals and other non-bilaterian organisms (electronic supplementary material, figures S5 and S6). While the potential link between genome size of eukaryotes and TEs has been demonstrated in arthropods and chordates (e.g. insects, myriapods, larvaceans and other chordates [27–29]), the genome of *Actinernus* sp. demonstrates that this association is phylogenetically more widespread, now including a cnidarian.

**Figure 2.**
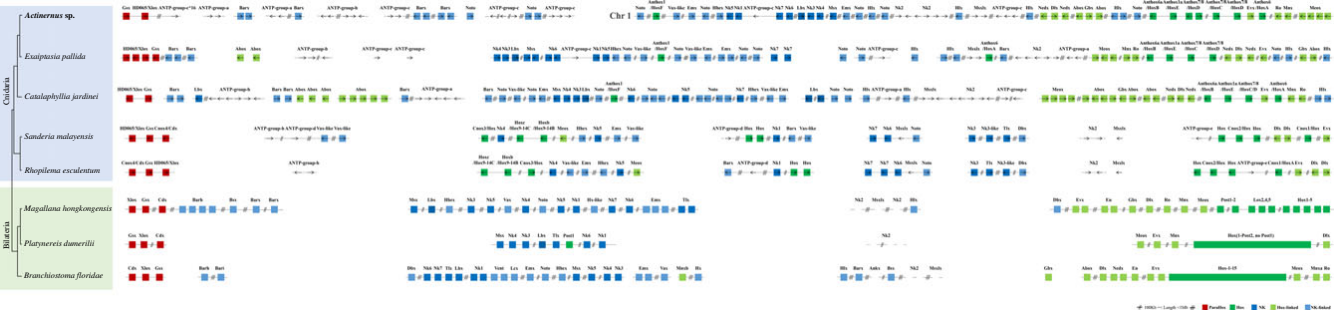
ANTP-class homeobox gene arrangement in high-quality cnidarian genomes compared with selected bilaterians. Chromosome 1 of *Actinernus* sp. is indicated with ‘Chr 1’. Double slash denotes genomic distance between 100 kb and 1 Mb; triple slash denotes genomic distance over 1 Mb. ParaHox, NK, NK-linked, Hox and Hox-linked genes (defined as previously described [16]) are coloured in red, blue, light blue, green and light green, respectively, while other genes are coloured in grey. Arrows inside gene boxes represent transcriptional orientation.

Homeobox genes are important developmental genes and can also serve as markers of large-scale genomic changes during metazoans evolution [16,30]. Previous studies suggested that a ProtoANTP gene underwent a series of tandem duplications to produce the Hox, ParaHox and NK cluster genes that at some point deep in animal ancestry existed as a ‘Mega-cluster’ of ANTP-class homeobox genes [17]. Genomic analyses have identified that cnidarians and bilaterians seemingly took different evolutionary pathways to their ANTP-class homeobox genes organization since their last common ancestor. Bilaterians tend to have a similar split of their ANTP-class gene families across approximately four distinct chromosomes, consisting of the Hox cluster genes with a handful of HoxL genes on one chromosome, the NK cluster genes and a few NKL genes on another, with the ParaHox cluster and the NK2 genes on two further distinct chromosomes [18,19,31–33]. In contrast, some cnidarians have been found to have mixtures of a small number of Hox, HoxL, NK and NKL genes dispersed around their respective genomes [34,35] (figure 2). To our surprise, we found the first case of the existence of the previously hypothetical mega-homeobox array of Hox cluster, HoxL, NK cluster and NKL genes in an extant species, on *Actinernus* chr 1 (figure 2; electronic supplementary material, S6 and figures S7–S10). Since chr 1 is the largest chromosome in the *Actinernus* genome, whether this genomic organization represents a primary linkage array directly descendant and retained from the cnidarian–bilaterian ancestor, or instead represents a secondary ‘coming together’ of these genes (e.g. via chromosome fusion events), remains to be established, for example, by further genome sequencing and linkage analyses in a wider diversity of species [16]. Nevertheless, the widespread linkage of Hox/HoxL with NK/NKL genes that is also found in other cnidarian species (figure 2) would imply that this *Actinernus* condition may well reflect the ancestral state.

Further homeodomain phylogenetic tree analyses also identified an anthozoan-specific group of ANTP-class genes, termed ANTP-group-c here that was expanded in *Actinernus* sp. (electronic supplementary material, figure S8). Twenty-four out of 28 of these *Actinernus* ANTP-group-c genes resulted from tandem duplications on four chromosomes based on the comparable patterns of conserved TEs around them (electronic supplementary material, figure S11). Interestingly, this preponderance of tandem duplications leading to the expansion of the complement of homeobox genes, as shown here in these ANTP-group-c genes, is consistent with the assumptions underpinning the Mega-cluster hypothesis discussed above, whereby ancestral precursors to the ANTP-class gene families are hypothesized to most likely have originated via tandem duplications. Whether this ANTP-class homeobox gene expansion of ANTP-group-c occurred more widely in deep-sea cnidarians remains to be tested.

**Figure 3.**
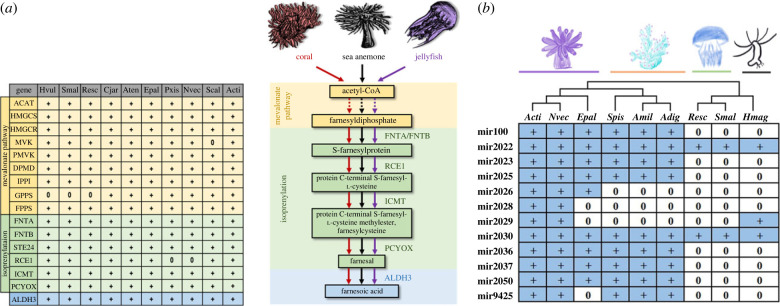
(*a*) Conserved genes in the sesquiterpenoid biosynthesis pathway among cnidarians and (*b*) conserved cnidarian microRNAs that are shared between at least two sea anemone species. ‘+’ and ‘0’ signs denote the presence and absence of genes/microRNAs in respective species. Species abbreviations: Acti, *Actinernus* sp.; Adig, *Acropora digitifera*; Amil, *Acropora millepora*; Aten, *Actinia tenebrosa*; Cjar, *Catalaphyllia jardinei*; Epal, *Exaiptasia pallida*; Hmag, *Hydra magnipapillata*; Hvul, *Hydra vulgaris*; Nvec, *Nematostella vectensis*; Pxis, *Paraphelliactis xishaensis*; Resc, *Rhopilema esculentum*; Scal, *Scolanthus callimorphus*; Smal, *Sanderia malayensis*; Spis, *Stylophora pistillata*.

Beyond the homeobox complement, genes for sesquiterpenoid hormone production such as farnesoic acid, methyl farnesoate and juvenile hormone were long thought to be confined to regulation of development and reproduction of insects and other arthropods [29,36,37]. Recently, genes for this pathway were unexpectedly revealed in jellyfish genomes [32]. Sesquiterpenoid hormone systems are now also known in other cnidarian genomes, and farnesoic acid has also been detected in jellyfish [34,35,38]. In the *Actinernus* sp. genome and transcriptomes, we found that genes were expressed throughout the whole sesquiterpenoid biosynthetic pathway, presumably leading to the *de novo* synthesis of farnesoic acid in this species as well (figure 3*a*; electronic supplementary material, S7 and figures S12 and S13). The identification of these genes implies that sesquiterpenoids are of widespread importance across the cnidarians, even in the deep-sea environment, where their precise function remains to be determined.

MicroRNAs are 21–23 nucleotides small RNAs that play important post-transcriptional gene regulation roles in animals. Previous studies have shown that cnidarians and bilaterians only share one conserved microRNA (miR-100) between them [32,34,35,39,40]. We produced small RNA transcriptomes for *Actinernus* sp. and annotated a total of 28 microRNAs in its genome (figure 3*b*, electronic supplementary material, S8). Among these annotated microRNAs, we identified 12 conserved microRNAs across different lineages of cnidarians, including miR-100, miR-2022, miR-2023, miR-2030, miR-2025, miR-2026, miR-2028, miR-2029, miR-2036, miR-2037, miR-2050 and miR-9425 (figure 3*b*; electronic supplementary material, figure S14). Taking *Actinernus* sp. as a reference point, we see a gradually nested pattern of these conserved microRNAs at successive nodes in the cnidarian phylogeny (figure 3*b*). Such a pattern would be consistent with them having been incorporated into the gene regulatory networks of functions that have progressively assembled at distinct nodes that represent successive ancestors within the cnidarians. In addition, independent expansions of microRNAs also happened in individual cnidarian lineages, illustrating the converse pattern to the widely conserved genes and demonstrating the divergent evolution of these ancient cnidarian lineages. MicroRNAs thus provide a mixed evolutionary pattern ranging from widely conserved to lineage-specific genes, but in total the cnidarian microRNA complements highlight the distinct nature of these animals from bilaterians.

**Figure 4.**
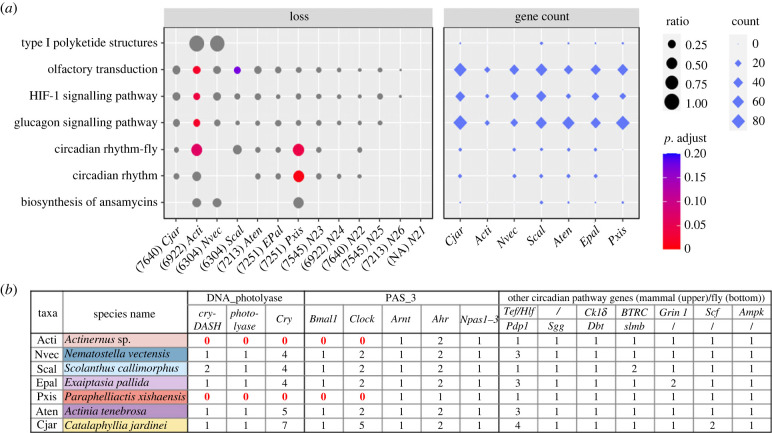
(*a*) Enriched KEGG pathways of gene family loss (left panel) and gene count in Hexacorallia taxa. The gene ratio was defined by the number of gene loss at each taxon/internode divided by the total number of genes in its ancestral state. *P*-value was calculated to test significantly over-represented genes using the hypergeometric model and was adjusted using the Benjamini and Hochberg method (BH) and (*b*) gene count table of circadian rhythm pathway and related gene families with DNA photolyase and PAS fold (PAS_3) domains.

Phylogenomic analyses using 332 single-copy orthologues suggested *Actinernus* diverged from the last common ancestor of itself and the sea anemones *Scolanthus callimorphus* and *Nematostella vectensis* about 250 Ma (electronic supplementary material, figure S15). Gene loss has been suggested as an important force in metazoan evolution [41], and our enrichment analyses identified the loss of circadian rhythm genes and DNA photolyases in both *Actinernus* sp. and another deep-sea anemone, *Paraphelliactis xishaensis* (figure 4; electronic supplementary material, S9 and figures S16–S19). Although the evidence of gene loss in *P. xishaensis* could be limited by the quality of the available draft genome [14], the high quality and physical continuity of this *Actinernus* sp. genome enabled us to carry out microsyntenic analyses to confirm the gene losses, including cryptochrome, cryptochrome-DASH, deoxyribodipyrimidine photolyase, brain and muscle Arnt-like protein-1 (*Bmal1*), and circadian locomotor output cycles kaput (*Clock*) genes (electronic supplementary material, figure S20). Evolution of morphology and physiology in concert with gene loss has been documented in multiple examples in metazoans, including changes to the pelvis and spines in stickleback fishes [42], eye degeneration and pigment losses in cavefish [43], visual adaptation and loss of photoreceptors and olfactory receptors in deep-sea fishes [44,45] and loss of circadian genes in a sub-surface blind centipede [46]. Taken together, the adaptations to the deep-sea environment found in this anemone include gene losses associated with processes that have become redundant in the darkness (e.g. circadian clock), demonstrating the dynamic nature of gene complement evolution in relation to ecological evolutionary adaptation.

This study has provided high-quality genomic and transcriptomic resources of a deep-sea cnidarian, *Actinernus* sp. Our analyses reveal a distinctive genome architecture spread over 15 chromosomes that includes a mega-array of ANTP-class homeobox genes that until now was only hypothesized to exist in ancient animal ancestors. In addition, patterns of gene losses such as those in the circadian clock genes coincide with living in the deep-sea habitat, and appear to be generally applicable across animals inhabiting this extreme environment.

## 3. Methods

### (a) Sample collection and genome sequencing

Six deep-sea anemone specimens were collected from Haima cold seep in the South China Sea at 1386 m depth during a HYDZ6-202005 cruise of the research vessel R/V Haiyangliuhao in September 2020. Samples were instantly frozen in liquid nitrogen and kept frozen during delivery and storage. The whole body was sent to Novogene (Hong Kong) to perform genomic DNA extraction. PacBio Biosciences HiFi circular consensus sequencing (CCS) library was constructed and sequenced by Novogene (Hong Kong).

### (b) Dovetail Omni-C library preparation and sequencing

For each Dovetail Omni-C library, chromatin was fixed in place with formaldehyde in the nucleus and then extracted. Fixed chromatin was digested with DNAse I, chromatin ends were repaired and ligated to a biotinylated bridge adapter followed by proximity ligation of adapter-containing ends. After proximity ligation, crosslinks were reversed and the DNA purified. Purified DNA was treated to remove biotin that was not internal to ligated fragments. Sequencing libraries were generated using NEBNext Ultra enzymes and Illumina-compatible adapters. Biotin-containing fragments were isolated using streptavidin beads before PCR enrichment of each library. The library was sequenced on an Illumina HiSeqX platform to produce an approximately 49× sequence coverage (electronic supplementary material, S2). Then HiRise was applied for reads with MQ > 50 for scaffolding.

### (c) mRNA and small RNA transcriptomes

mRNA and small RNA were extracted from tentacle and trunk tissues of two individuals using TRIzol (Ambion) and the miRVana Isolation kit, respectively. The concentrations were measured using a NanoDrop One/One C Microvolume UV Spectrophotometer (Thermo Fisher Scientific, Fitchburg, WI, USA). Agarose gel electrophoresis was used to assess the quality. Samples were stored at −80°C and sent to Novogene (Hong Kong) for transcriptome (Novaseq, PE150 platform for strand-specific library construction) and small RNA library (Novaseq, 50SE platform for strand-specific library construction) sequencing (electronic supplementary material, S2).

### (d) Genome assembly and scaffolding with HiRise

The *de novo* genome assembly was carried out with Hifiasm [47], and haplotypic duplication was identified and removed with purge_dups based on the depth of HiFi reads [48]. The primary genome assembly and Dovetail Omni-C library reads were then used as input data for HiRise [49]. Dovetail Omni-C library sequences were aligned to the draft input assembly using bwa (https://github.com/lh3/bwa). The separations of Dovetail Omni-C read pairs mapped within draft scaffolds were analysed by HiRise to produce a likelihood model for genomic distance between read pairs, and the model was used to identify and break putative misjoins, to score prospective joins and make joins. The mitochondrial genome of *Actinernus* sp. was assembled by MitoHiFi [50] (v.2.2, https://github.com/marcelauliano/MitoHiFi) using PacBio HiFi reads and *Macrodactyla doreensis* (NC_066448.1) as the mitochondrial reference sequence.

### (e) Gene model prediction

Gene model annotations were processed as previously described [32]. In brief, gene models were trained and predicted using funannotate (v.1.8.9). Protein-coding genes were searched with BLASTp against the nr and swissprot databases by diamond (v.0.9.24) with parameters ‘—more-sensitive—*e*-value 1 × 10^−3^,’ and mapped by HISAT2 v.2.1.0 with transcriptome reads from the tentacle and columnar trunk tissues. Gene models with no similarities to any known protein and no messenger RNA support were removed from the final version, filtered by CGAT [51], and only the longest genes were retained for subsequent analyses. Repetitive elements were identified using the TE annotation pipeline Earl Grey [52] as described previously [53].

### (f) Macrosynteny analysis

Orthologous gene pairs were retrieved from reciprocal BLASTp hits (*e*-value 1 × 10^−5^) between *Actinernus* sp. and each taxon in the orthology analysis. Macrosynteny was inferred by comparing *Actinernus* sp. with four chromosome-level Hexacorallia genomes (*S. callimorphus*, *N. vectensis*, *Exaiptasia pallida* and *Catalaphyllia jardinei*) using MCScanX under default parameters [54] and visualized with TBtools v.1.055 [55]. *DupGen*_*finder* [56] was used to parse MCScanX results and classify duplicated genes in *Actinernus* sp. into self-syntenic duplications (originally whole-genome duplication), tandem duplications, proximal duplications, transposed duplications and dispersed duplications using the four chromosome-level Hexacorallia genomes as outgroups. Further, reciprocal best hits from the highest BLASTp bit-score between *Acinernus* sp. and the four Hexacorallia genomes and five other outgroups (*Rhopilema esculentum*, *Sanderia malayensis*, *Hydra vulgaris*, *Ephydatia muelleri* and *Hormiphora californensis*) were used to generate Oxford dot plots as described [29,57].

### (g) Annotation of sesquiterpenoid pathway genes

Gene sequences involved in the sesquiterpenoid biosynthetic pathway were retrieved from *Nematostella* and previous studies [32] and were used for searching against the gene models and genome using BLASTP and TBLASTN, respectively. Putatively identified orthologues with a threshold of *e*-value equal to 10^−3^ were tested by reciprocal searches in the NCBI nr database using BLASTP. The gene expression profile of the sesquiterpenoid biosynthetic pathway was visualized with a heatmap of trimmed mean of *M*-values normalized counts from the tentacle and trunk transcriptomes under log_2_ scale in TBtools [55].

### (h) microRNA annotation

Cnidarian microRNAs were annotated as previously described [32]. Adaptor sequences were trimmed from small RNA sequencing reads and reads with Phred quality scores less than 20 were removed. Processed reads of lengths between 18 bp and 27 bp were then mapped to the genomes using the mapper.pl module of the mirDeep2 package [58]. To identify known miRNAs, the predicted deep-sea anemone microRNA hairpins were compared against metazoan microRNA precursor sequences from miRbase [59] using BLASTN (*e*-value < 1 × 10^−2^). miRNAs with no significant sequence similarity to any of the miRNAs in miRBase were checked manually. Novel microRNAs were defined as those that fulfilled the criteria of microRNAs in MirGeneDB [60–62]. In addition, precursor sequences of microRNAs from other sea anemones in previous studies [40,63,64] were also used to carry out BLASTN searches for identification of any missed microRNA annotations. Multiple alignments of conserved miRNAs were carried out by MEGA7 [65].

### (i) Annotation of homeobox genes

Potential homeobox genes were identified by searching with homeodomain sequences from *N. vectensis* [66], *Branchiostoma floridae*, *Drosophila melanogaster* (retrieved from HomeoDB2 [67] in the *Actinernus* sp. genome and previous published high-quality cnidarian genomes [14,31,32,34,35,57,68–73]) using tBLASTN (electronic supplementary material, S4). NCBI CD-Search [74] was then used to validate the presence of homeodomains in the retrieved sequences. Identification of each putative gene was tested by comparison to sequences confirmed in the NCBI nr database and previous studies [32,67,75–86] using BLASTx and BLASTp, phylogenetic analysis and syntenic analysis (electronic supplementary material, S4). Syntenic relationships between *E. pallida*, *N. vectensis*, *Acropora millepora*, *R. esculentum* and *H. vulgaris* were computed using MCScanX [54] with default parameters. MG2C [87] was used to visualize the mega-array of homeodomain genes on a chromosome map of Chr 1 in *Actinernus* sp.

### (j) Orthology and gene family evolution analysis

The gene orthology of the longest gene transcripts from six actiniarians and nine outgroup taxa was inferred by OrthoMCL [88]. Three-hundred and thirty-two single-copy genes were aligned by MAFFT v.7.271 [89], trimmed with trimAl v.1.4.rev15 [90] and concatenated to construct a species phylogenetic tree using RAxML v.8.2.9 [91] with the PROTGAMMAILGF model and 1000 bootstrap replicates. BEAST v.2.6.2 [92] was employed to infer the divergence times with a relaxed clock rate and calibrated Yule model with gamma prior distribution. The calibration points were retrieved from the TimeTree web database (http://timetree.org): *Salpingoeca rosetta*–*H. californensis* (mean 908 Ma, sigma: 30), *H. californensis–E. muelleri* (mean 851 Ma, sigma: 35), *Trichoplax adhaerens–H. vulgaris* (mean 661, sigma: 27), *H. vulgaris–R. esculentum* (581 Ma, sigma: 30) and *C. jardinei–Actinia tenebrosa* (mean 532, sigma: 25). The first 30% of the MCMC sampling was discarded as burn-in. The resulting species tree was used as the input tree for CAFE analysis [93], where multiple lambdas were used to infer the birth–death rate for four groups, namely Choanozoa-Placozoa, Hydrozoa-Scyphozoa, Scleractinia and Actinaria.

### (k) Functional enrichment analysis

Functional annotations were processed in each analysed proteome with eggnog [94]. Orthologous groups were assigned for annotation terms from Gene Ontology (GO), EuKaryotic Orthologous Groups (KOG), Kyoto Encyclopedia of Genes and Genomes (KEGG) and KEGG Orthology (KO) when they were observed in genes within an orthologous group. Functional enrichment was tested as previously described [29,38]. Enrichment was tested with the function ‘compareCluster()’ from R package ‘clusterProfiler’ v.3.16.1 [95]. For enriched terms that resulted from gene family gain, their significance was determined with *p*-value cut-off of 0.05 and *q*-value cut-off of 0.2, according to adjustment of *p*-value using the Benjamini and Hochberg (BH) method. To validate gene family gains, protein family searches using HMMER [96] (v.3.3.1; cut-off *e*-value < 10^−5^) followed by confirmation with Reverse Position-Specific BLAST (RPS-BLAST) with the NCBI's Conserved Domain Database (CDD) [97] were used to generate a gene count table. For enriched terms that resulted from gene loss, as statistical significance may not be applicable, they were manually checked by sorting from high to low (ratio of 1.0 conveys complete loss of a gene family). The absence of gene family was further validated with tBLASTn and BLASTp as well as protein family (HMMER) searches [96,98] of reference protein sequences in the genome and proteome, respectively. Microsynteny of neighbouring conserved genes at a loss site was inspected by comparing with other Hexacorallia genomes using MCScanX [54], followed by visualization in the *gggenomes* package [99] in *R* v.4.1.1 [100].

## Data Availability

The Whole Genome Shotgun project was deposited at DDBJ/ENA/GenBank under the accession JAOWCC000000000. The raw reads generated in this study were deposited to the NCBI database under the BioProject accessions PRJNA889077. The genome annotation files were deposited in Figshare: https://figshare.com/s/162e2b28fc843c840a65. Files can also be found here: https://datadryad.org/stash/share/8KypWmpBYJgpPXhWPE5PsO4citcscg4IJShJ61VQgI4 [101]. The data are provided in the electronic supplementary material [102].
